# Supplementary material for: Mucosal vaccination clears Clostridioides difficile colonization
Source: Nature. 2026 Feb 18;652(8112):1289–97. doi: 10.1038/s41586-026-10138-x (PMC13128438; doi:10.1038/s41586-026-10138-x)
Supplement: Supplementary file 1 — Further information: antigen quality control for FlgGEK and TcdB2 mutants; raw gel images corresponding to Extended Data Fig. 4; and flow cytometry gating schemes. [file 41586_2026_10138_MOESM1_ESM.pdf]

nature portfolio

https://doi.org/10.1038/s41586-026-10138-x

Supplementary information

Mucosal vaccination clears *Clostridioides difficile* colonization

In the format provided by the authors and unedited

Mucosal vaccination clears *Clostridioides difficile* colonization

Audrey K. Thomas, F. Christopher Peritore-Galve, Alyssa G. Ehni, Bruno B.C. Lança, Jonathan Coggin, Eric J. Brady, Sandra M. Yoder, Rebecca Shrem, Rubén Cano Rodriguez, Heather K. Kroh, Katherine N. Gibson-Corley, M. Kay Washington, Danyvid Olivares-Villagómez, C. Buddy Creech, Maribeth R. Nicholson, Benjamin W. Spiller, & D. Borden Lacy

Supplementary Antigen Quality Control

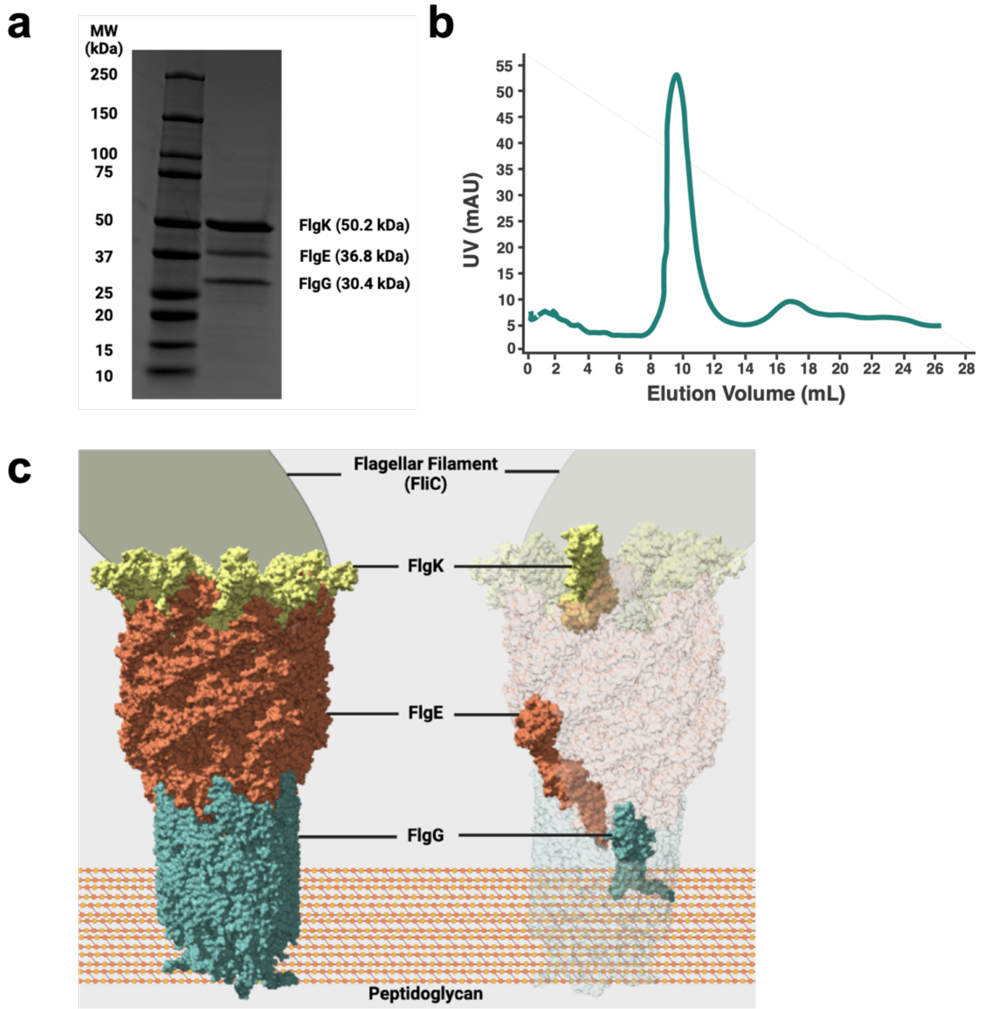

FlgG, FlgE, and FlgK co-purify when mixed and form a ternary complex, FlgGEK.
(a) Annotated SDS-PAGE gel of FlgGEK complex. **(b)** Chromatogram off an S200 sizing column of FlgGEK complex, which elutes at a volume between 8-12 mL. **(c)** FlgGEK representative model based off *Campylobacter jejuni* FlgG and FlgE (PDB: 7CGO) and FlgK (5XBJ). Made in ChimeraX and BioRender.

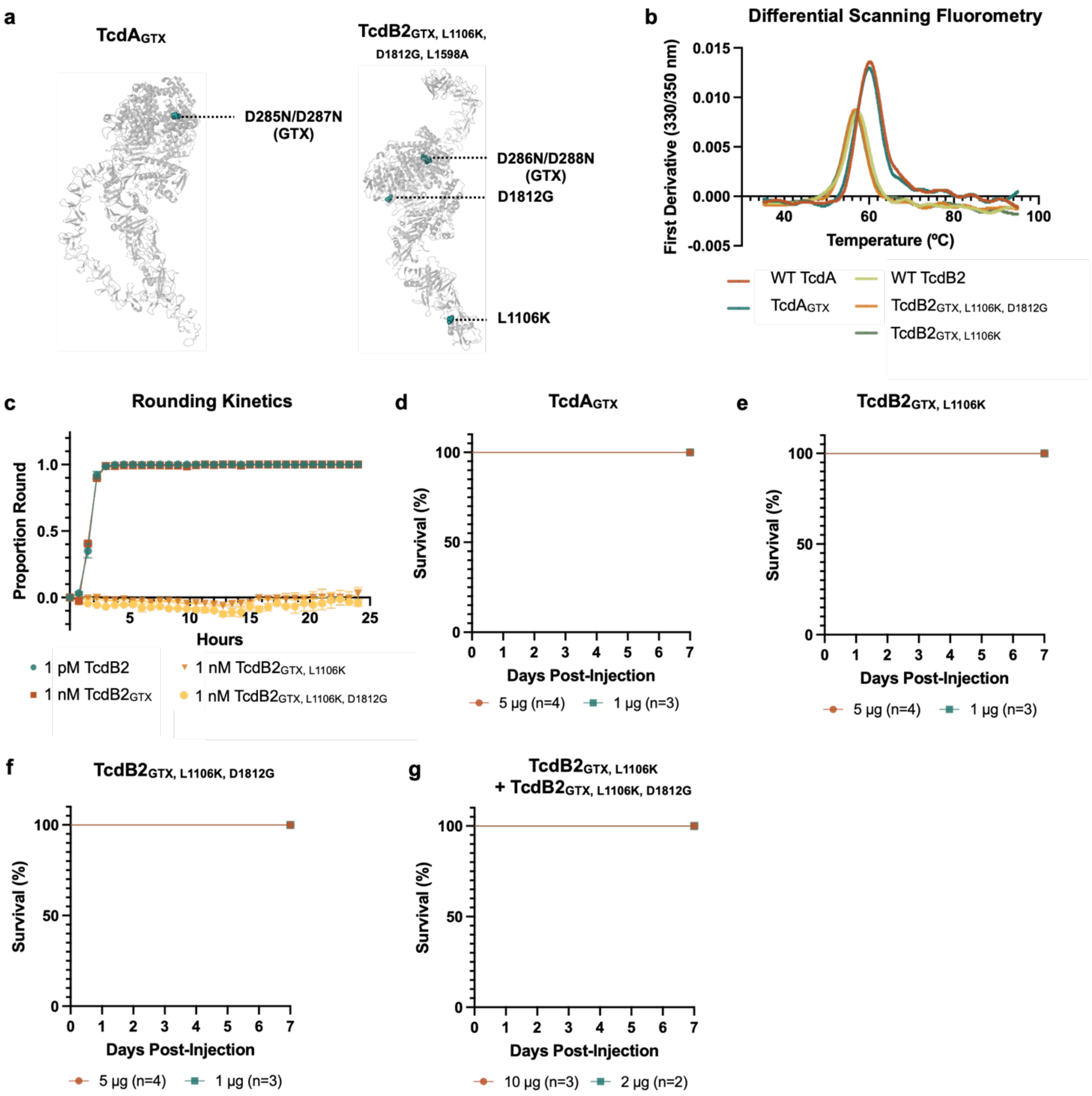

Testing *C. difficile* point-mutant toxins for activity *in vitro* and *in vivo*.
(a) Structures of TcdA (7POG) and TcdB2 (6OQ5) are shown with the location of point mutations indicated in teal. $TcdA_{GTX}$ (D285N/D287N) and TcdB2 (D286N/D288N, L1106K, D1812G), figures created in Pymol. **(b)** 60 µg of wild-type or point mutants of TcdA and TcdB2 were thermally denatured and the ratio of protein intrinsic fluorescence (first derivative) was captured. Similar first derivative peaks indicate similar denaturation patterns and suggest native folding. **(c)** Vero-GFP cells were incubated for 12 hours with 1 pM TcdB2 or 1 nM of various TcdB point mutants, and images were taken in 30 min intervals. Rounded cells indicate cytopathic effects of the *C. difficile* toxins. Performed in technical triplicate. **(d to g)** Survival curves for mice intraperitoneally-injected with various quantities of mutated TcdA and TcdB toxins. n = 2-4 per group.

<u>Supplementary Gel Images (from Extended Figure 4)</u>

<u>Note:</u> Aside from molecular weight (MW) ladders (in base pairs, bp), each lane represents a unique mouse. Gels on the left are original, unedited images; gels on the right are enhanced. The white dotted box indicates where the image was cropped. Cropped and enhanced gels are below for each group. RI, rectal instillation; IP, intraperitoneal injection.

<u>Colon PCR Gels</u>

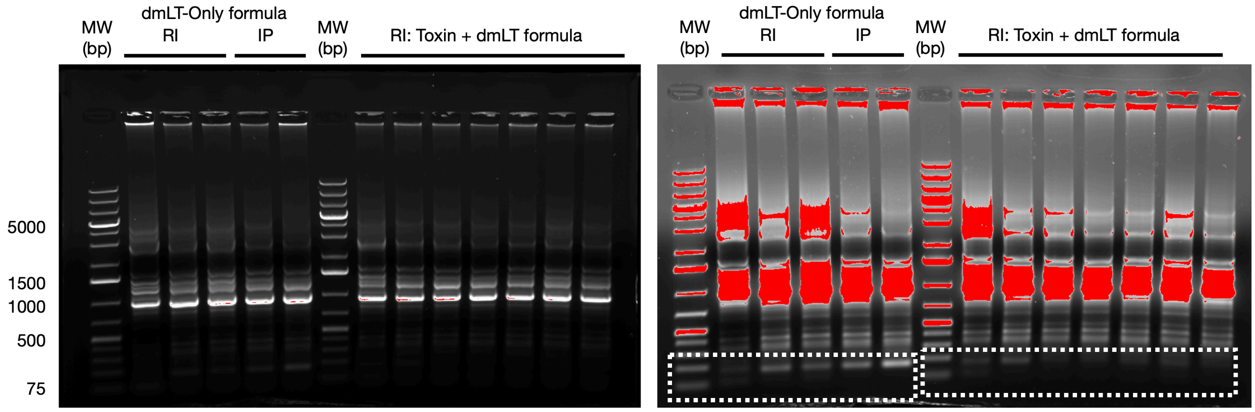

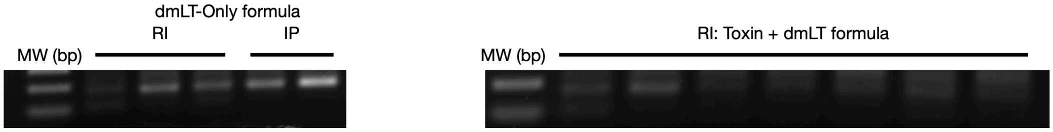

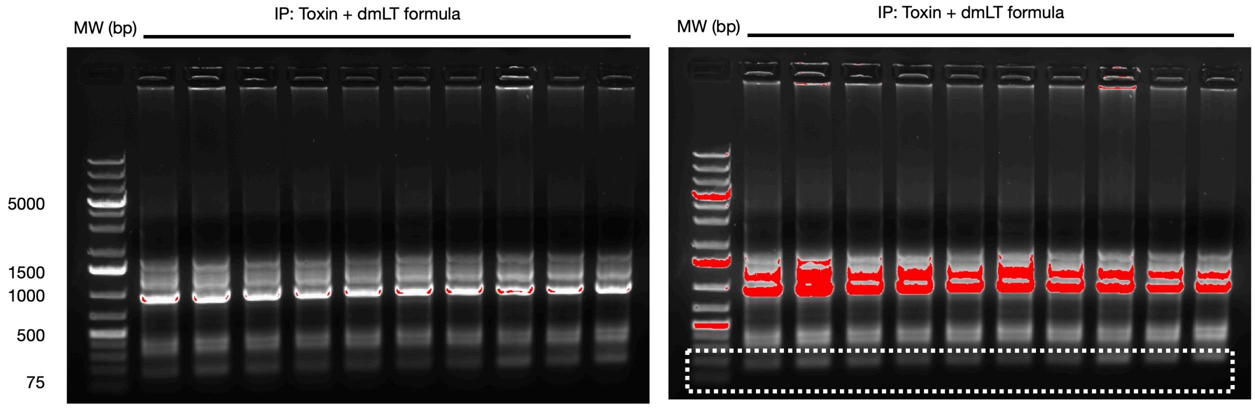

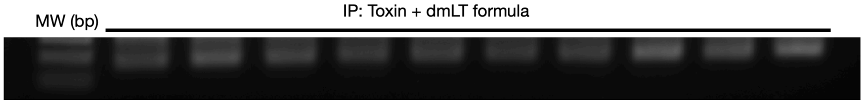

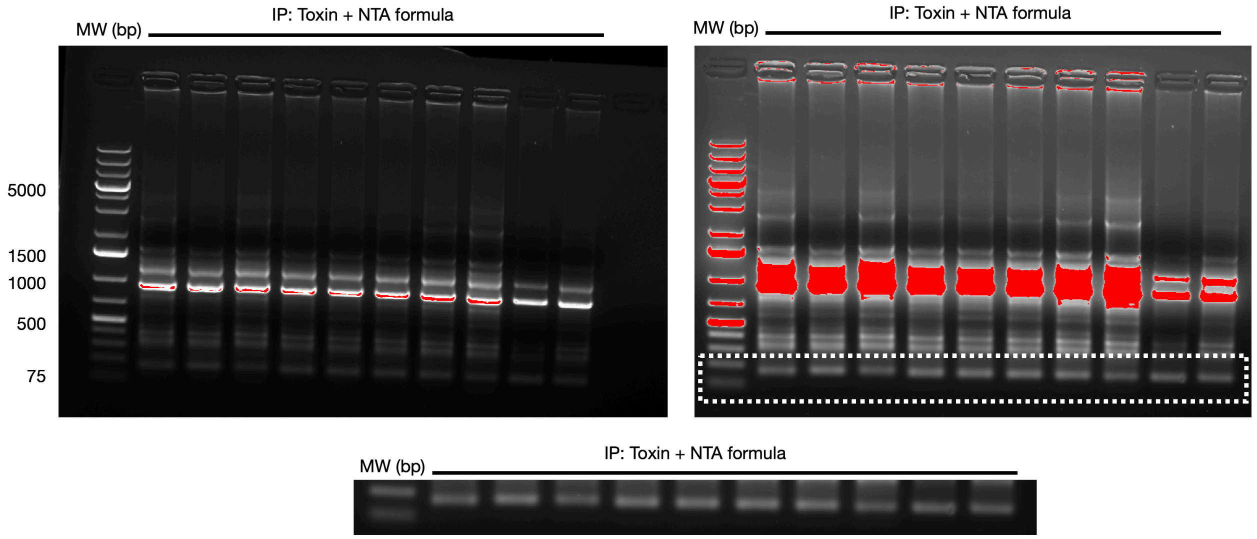
MW (bp)
IP: Toxin + NTA formula
5000
1500
1000
500
75
MW (bp)
IP: Toxin + NTA formula
MW (bp)
IP: Toxin + NTA formula

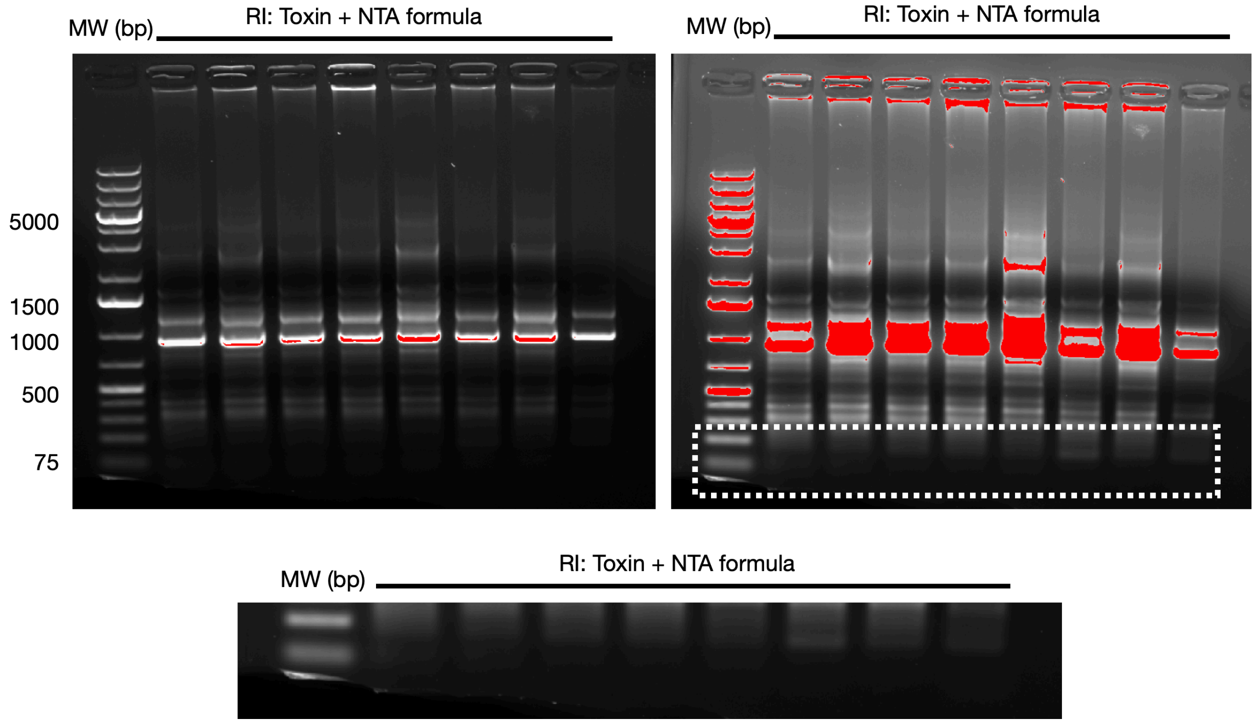
MW (bp)
RI: Toxin + NTA formula
5000
1500
1000
500
75
MW (bp)
RI: Toxin + NTA formula
MW (bp)
RI: Toxin + NTA formula

Cecum PCR Gels

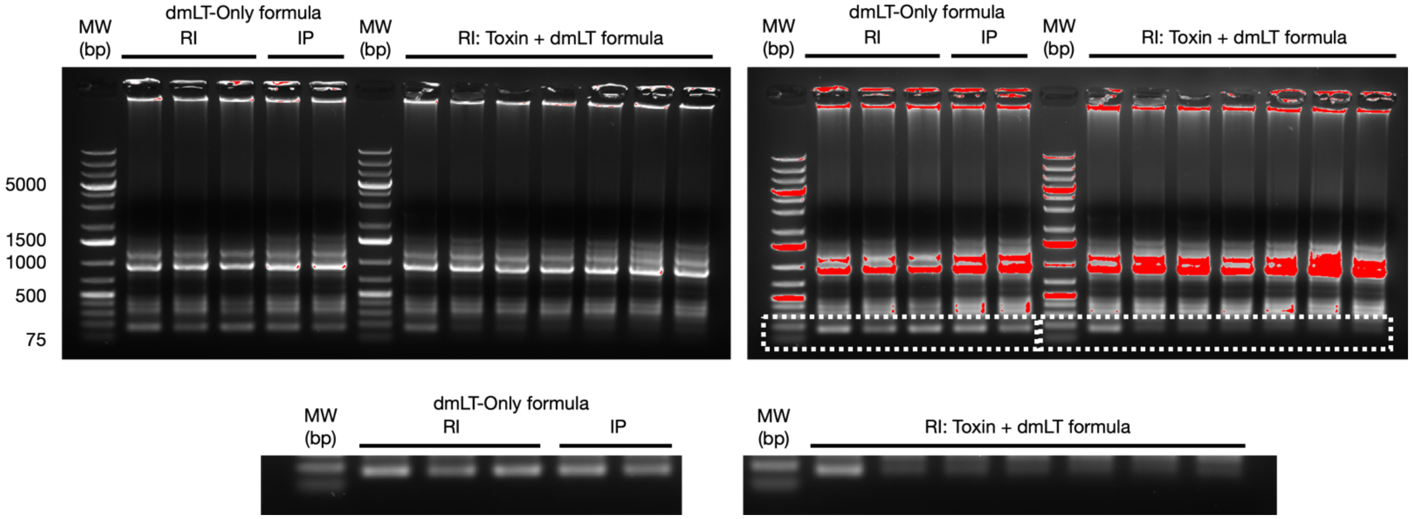

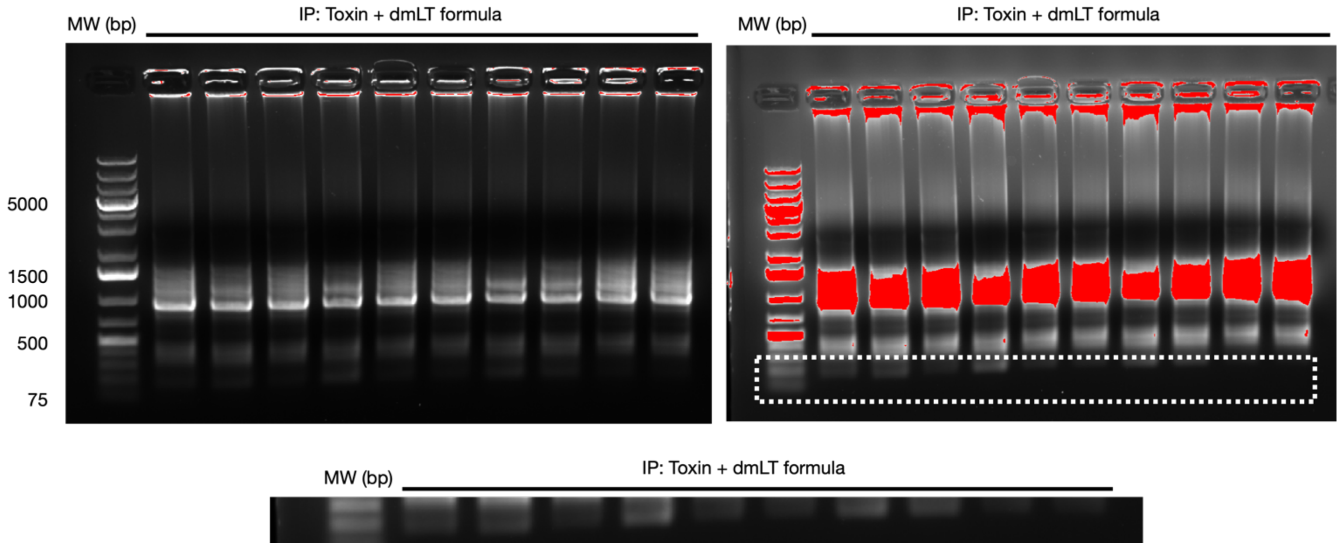

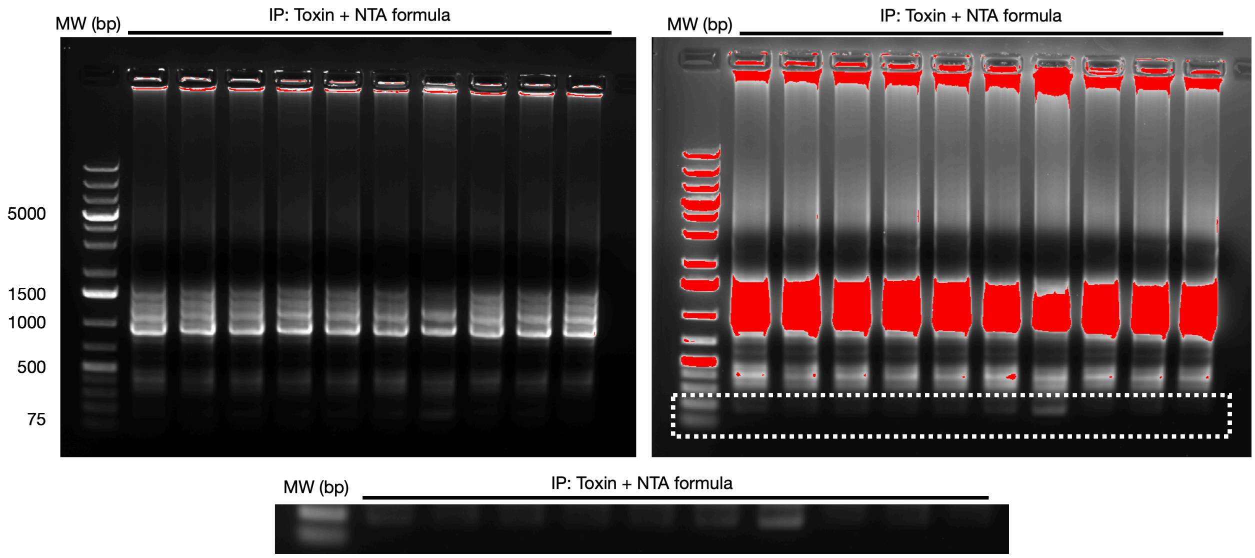
MW (bp)
IP: Toxin + NTA formula
5000
1500
1000
500
75
MW (bp)
IP: Toxin + NTA formula
MW (bp)
IP: Toxin + NTA formula

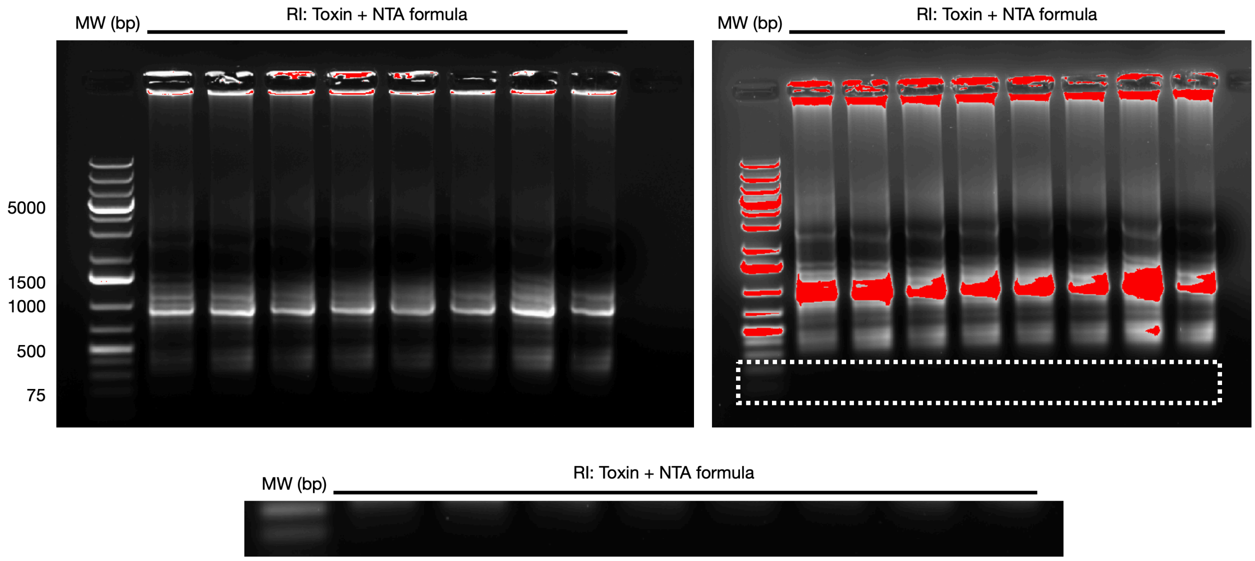
MW (bp)
RI: Toxin + NTA formula
5000
1500
1000
500
75
MW (bp)
RI: Toxin + NTA formula
MW (bp)
RI: Toxin + NTA formula

Supplementary Flow Cytometric Gating Schemes

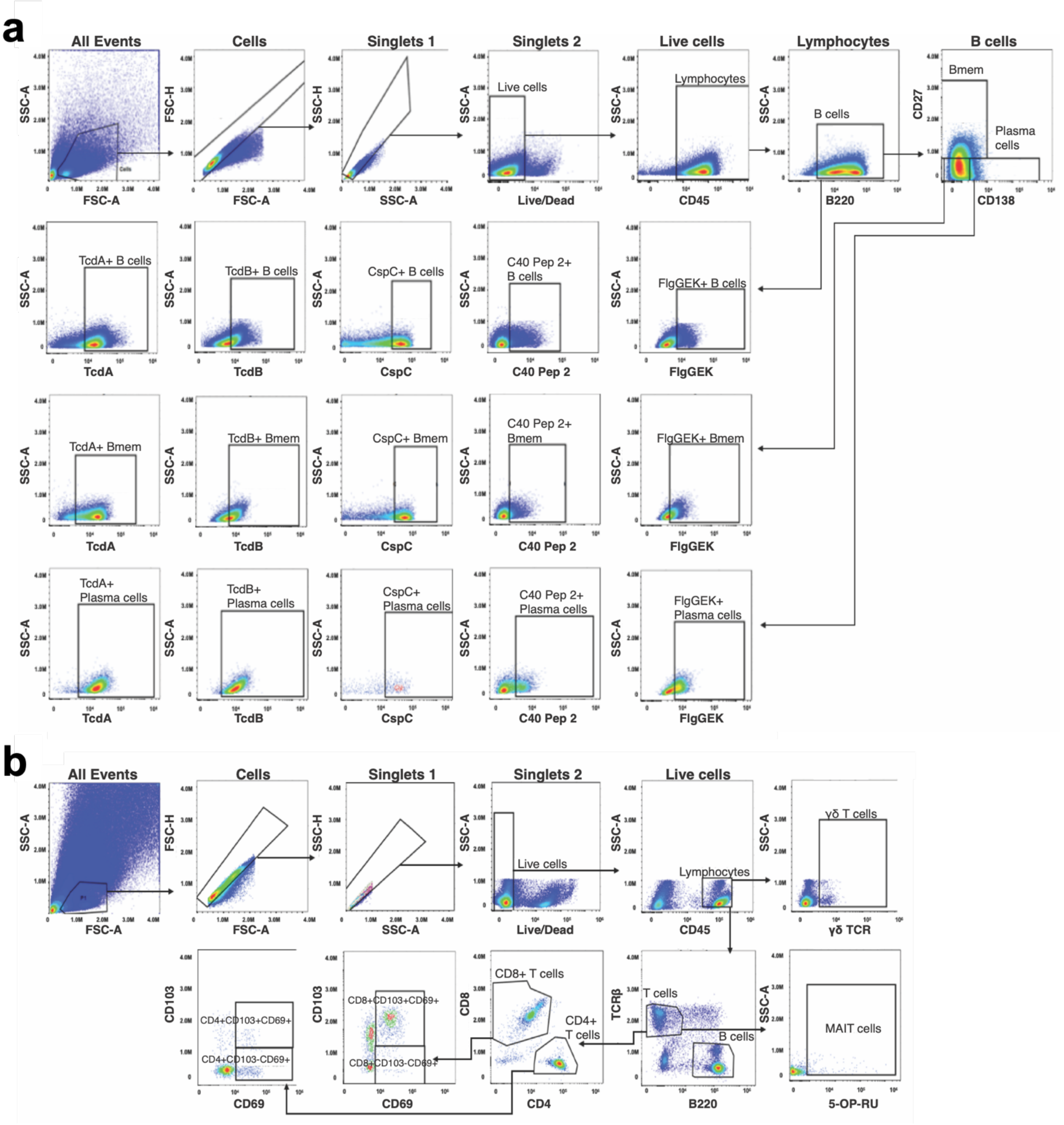

Gating strategies for adaptive immune responses.
(a) Gating strategies to identify antigen-specific and polyclonal B cells in spleens and mesenteric lymph nodes. **(b)** Gating strategies to identify CD4+ and CD8+ T cells in colonic intraepithelial and lamina propria populations. Fluorophores used are listed in the Materials and Methods. C40 Pep 2, C40 peptidase 2; MAIT, mucosal-associated invariant T cells.
