# Supplementary material for: Mucosal vaccination clears Clostridioides difficile colonization
Source: Nature. 2026 Feb 18;652(8112):1289–97. doi: 10.1038/s41586-026-10138-x (PMC13128438; doi:10.1038/s41586-026-10138-x)
Supplement: Supplementary file 2 — Reporting Summary [file 41586_2026_10138_MOESM2_ESM.pdf]

nature portfolio

Corresponding author(s): Wei Guo, Ph.D.

Last updated by author(s): Jan 12, 2026

Reporting Summary

Nature Portfolio wishes to improve the reproducibility of the work that we publish. This form provides structure for consistency and transparency in reporting. For further information on Nature Portfolio policies, see our Editorial Policies and the Editorial Policy Checklist.

Statistics

For all statistical analyses, confirm that the following items are present in the figure legend, table legend, main text, or Methods section.

n/a	Confirmed	
☐	☒	The exact sample size (*n*) for each experimental group/condition, given as a discrete number and unit of measurement
☐	☒	A statement on whether measurements were taken from distinct samples or whether the same sample was measured repeatedly
☐	☒	The statistical test(s) used AND whether they are one- or two-sided<br>*Only common tests should be described solely by name; describe more complex techniques in the Methods section.*
☐	☒	A description of all covariates tested
☒	☐	A description of any assumptions or corrections, such as tests of normality and adjustment for multiple comparisons
☐	☒	A full description of the statistical parameters including central tendency (e.g. means) or other basic estimates (e.g. regression coefficient) AND variation (e.g. standard deviation) or associated estimates of uncertainty (e.g. confidence intervals)
☐	☒	For null hypothesis testing, the test statistic (e.g. *F*, *t*, *r*) with confidence intervals, effect sizes, degrees of freedom and *P* value noted<br>*Give P values as exact values whenever suitable.*
☒	☐	For Bayesian analysis, information on the choice of priors and Markov chain Monte Carlo settings
☒	☐	For hierarchical and complex designs, identification of the appropriate level for tests and full reporting of outcomes
☐	☒	Estimates of effect sizes (e.g. Cohen's *d*, Pearson's *r*), indicating how they were calculated

Our web collection on statistics for biologists contains articles on many of the points above.

Software and code

Policy information about availability of computer code

Data collection	Flowjo V10, GraphPad Prism v.8.0, NIS-Elements Advanced Research software Version 4.50,ImageJ v1.54h
Data analysis	Statistical analysis were performed on GraphPad Prism v.8.0. Flowcytometry data were analyzed on FlowJo software package (Flowjo V10).

For manuscripts utilizing custom algorithms or software that are central to the research but not yet described in published literature, software must be made available to editors and reviewers. We strongly encourage code deposition in a community repository (e.g. GitHub). See the Nature Portfolio guidelines for submitting code & software for further information.

Data

Policy information about availability of data

All manuscripts must include a data availability statement. This statement should provide the following information, where applicable:

- Accession codes, unique identifiers, or web links for publicly available datasets
- A description of any restrictions on data availability
- For clinical datasets or third party data, please ensure that the statement adheres to our policy

The data that support the findings of this study are available within the paper and its supplementary Information files. RNA-seq data can be accessed through GSE302643. The data that support the findings of this study will be available from the corresponding author upon reasonable request.

Research involving human participants, their data, or biological material

Policy information about studies with human participants or human data. See also policy information about sex, gender (identity/presentation), and sexual orientation and race, ethnicity and racism.

Reporting on sex and gender	Blood samples from healthy male and female individuals were used in human macrophage experiments. There was no specific preference of covariate-relevant population characteristics for these individuals.<br><br>Paraffin blocks of tumor samples from 37 melanoma patients treated with Pembrolizumab at Hospital of University of Pennsylvania were used for immunofluorescence.
Reporting on race, ethnicity, or other socially relevant groupings	Information for melanoma is shown in Supplementary Table 2.
Population characteristics	Information for melanoma is shown in Supplementary Table 2.
Recruitment	Healthy individuals were recruited by by the Human Immunology Core at the University of Pennsylvania, with no specific selection basis for blood donation used for human macrophage isolation.<br><br>All melanoma patients were seen at Hospital of University of Pennsylvania during their clinical care. There was no selection bias.
Ethics oversight	Blood samples from human healthy donors were collected by the Human Immunology Core at the University of Pennsylvania with the approval from the ethics committee and institutional review board. Written consent was obtained from each healthy donor before blood collection. All experiments involving blood samples from healthy donors were performed in accordance with relevant ethical regulations.<br><br>All studies were conducted under protocols approved by University of Pennsylvania. All melanoma patients or families provided informed consent for research use of biospecimens and clinical data under an institutional approved protocol (IRB #703001).

Note that full information on the approval of the study protocol must also be provided in the manuscript.

Field-specific reporting

Please select the one below that is the best fit for your research. If you are not sure, read the appropriate sections before making your selection.

☒ Life sciences ☐ Behavioural & social sciences ☐ Ecological, evolutionary & environmental sciences

For a reference copy of the document with all sections, see nature.com/documents/nr-reporting-summary-flat.pdf

Life sciences study design

All studies must disclose on these points even when the disclosure is negative.

Sample size	For mouse studies, the sample size (n≥5 /group) was determined based on our previous experience with the models to provide sufficient statistical power (Chen et al., Nature 2018; Zhong et al. Nature Cancer, 2025;Zhong et al. Cancer Research, 2023; Zhang et al, Dev Cell, 2022). For human studies, the sample size was determined based on previous experience with the models to provide sufficient statistical power (Chen et al., Nature 2018;Moshe Sade-Feldman., Cell 2018).
Data exclusions	No data were excluded from analysis.
Replication	Figure legends describe the number of repeats for the experiments.
Randomization	Mice and other samples in experiments were allocated randomly to each treatment group.
Blinding	All experiments were performed in a blinded fashion. Downstream analyses of samples (immunofluorescence staining and flow cytometry) were performed in a blinded fashion; the individuals performing the assays were not aware of the treatment groups until data analyses were completed. For experiments other than those involving animals, the investigators were blinded to group allocation during data collection and/or analysis.

Reporting for specific materials, systems and methods

We require information from authors about some types of materials, experimental systems and methods used in many studies. Here, indicate whether each material, system or method listed is relevant to your study. If you are not sure if a list item applies to your research, read the appropriate section before selecting a response.

Materials & experimental systems

n/a	Involved in the study	
☐	☒	Antibodies
☐	☒	Eukaryotic cell lines
☒	☐	Palaeontology and archaeology
☐	☒	Animals and other organisms
☐	☒	Clinical data
☒	☐	Dual use research of concern
☒	☐	Plants

Methods

n/a	Involved in the study	
☒	☐	ChIP-seq
☐	☒	Flow cytometry
☒	☐	MRI-based neuroimaging

Antibodies

Antibodies used

Anti-human GM-CSFRα Cell Signaling Technology WB IF 1:1000 for WB;
1:100 for IP Cat#: 69817
Anti-mouse GM-CSFRα Biorbyt WB 1:500 Cat#: orb256474
Anti-M-CSFR Cell Signaling Technology WB 1:1000 Cat#: 3152
Anti-phospho-JAK2 Cell Signaling Technology WB 1:1000 Cat#: 3771
Anti-total JAK2 Cell Signaling Technology WB 1:1000 Cat#: 3230
Anti-phospho-STAT5 Cell Signaling Technology WB 1:1000 Cat#: 4322
Anti-total STAT5 Cell Signaling Technology WB 1:1000 Cat#: 94205
Anti-phospho-NF-κB Cell Signaling Technology WB 1:1000 Cat#: 3033
Anti-total NF-κB Cell Signaling Technology WB 1:1000 Cat#: 8242
Anti-β-actin Cell Signaling Technology WB 1:1000 Cat#: 4967
Anti-HRS Cell Signaling Technology WB 1:1000 Cat#: 15087S
Anti-HRS (phospho-Y216) Invitrogen WB 1:500 Cat#: PA5-114579
Anti-HRS (phospho-Y216) Biorbyt IF; IP 1:50 Cat#: orb644733
Anti-phospho-Src (Tyr416) Cell Signaling Technology WB 1:1000 Cat#: 2101
Anti-Src Cell Signaling Technology WB 1:1000 Cat#: 2109
Anti- DYKDDDDK (Flag) Cell Signaling Technology WB; IP 1:1000 for WB; 1:50 for IP Cat#: 14793
Mouse IgG isotype control BioLegend Blocking 10 μg/ml Cat#: 401404
Rat IgG isotype control Bio X Cell Blocking 10 μg/ml Cat#: BE0090
Anti-human M-CSFR BioLegend Blocking 10 μg/ml Cat#: 347302
Anti-mouse M-CSFR BioLegend Blocking 10 μg/ml Cat#:135502
Anti-human GM-CSFR BioLegend Blocking 10 μg/ml Cat#: 305902
Anti-mouse GM-CSFR Invitrogen Blocking
FCM 10 μg/ml for blocking
1:50 for FCM Cat#: MA5-23918
Anti-active Caspase-3 BD Biosciences FCM 1: 50 Cat#: 560901
Anti-active Caspase-3 BD Biosciences FCM 1: 50 Cat#: 570179
Anti-mouse Ki-67 BioLegend FCM 1: 50 Cat#: 652420
Anti-mouse Granzyme B eBioscience FCM 1: 50 Cat#: 12-8898-82
Anti-mouse CD8 BioLegend FCM 1: 100 Cat#: 126610
Anti-mouse CD3 BioLegend FCM 1: 100 Cat#:100228
Anti-mouse CD8 Cell Signaling Technology IF 1: 100 Cat#: 98941
Anti-mouse CD45.2 BioLegend FCM 1: 100 Cat#: 109835
Anti-mouse CD45.1 BioLegend FCM 1: 100 Cat#: 110735
Anti-human MHC-II BioLegend FCM 1: 100 Cat#: 327010
Anti-human CD86 BioLegend FCM 1: 100 Cat#: 374204
Anti-human CD206 BioLegend FCM 1: 100 Cat#: 321124
Anti-human CD163 BioLegend FCM 1: 100 Cat#: 333606
Anti-human MHC-II BioLegend FCM 1: 100 Cat#: 980414
Anti-mouse MHC-II BioLegend FCM 1: 100 Cat#: 107616
Anti-mouse CD86 BioLegend FCM 1: 100 Cat#: 105125
Anti-mouse CD206 BioLegend FCM 1: 100 Cat#: 141720
Anti-mouse CD163 BioLegend FCM 1: 100 Cat#: 111804
Anti-human CD63 Abcam WB 1: 1000 Cat#: ab134045
Anti-human CD63 Abcam IF 1: 200 Cat#: ab8219
Anti-human LAMP1 Cell Signaling Technology IF 1: 200 Cat#: 15665
Anti-Mouse IgG, F(ab')2 fragment specific Jackson ImmunoResearch FCM 1: 100 Cat#: 115-475-072
Anti-Mouse IgG (FITC), F(ab')2 fragment specific Jackson ImmunoResearch FCM 1: 100 Cat#: 109-096-006
Anti-Mouse IgG (APC), F(ab')2 fragment specific Jackson ImmunoResearch FCM 1: 100 Cat#: 115-136-072
Anti-EGFP Cell Signaling Technology WB 1: 1000 Cat#: 2956

Validation

All antibodies were verified by the supplier and each lot has been quality tested. All the antibodies used are from commercial sources and have been validated by the vendors. Validation data are available on the manufacturer's website.

Eukaryotic cell lines

Policy information about cell lines and Sex and Gender in Research

Cell line source(s)	The murine PDAC cell line 4662 cells were obtained as previously described (PMID: 25979873, PMID: 37115855), Melanoma B16-F10 cells (Cat#: CRL-6475);293T (Cat#: CRL-3216) were originally obtained from ATCC. WM9 cells, WM35 cells, the human leukocyte antigen (HLA)-matched cytotoxic T cells to WM35 cells and B16-OVA cells were generated as previously described (PMID: 37805922, PMID: 30089911); 4662-OVA cells were generated as previously described (PMID: 27642636). The cells were passaged for less than 1 month.
Authentication	A short tandem repeat DNA profiling method was used to authenticate the cell lines and the results were compared with reference database.
Mycoplasma contamination	All cells were regularly tested for Mycoplasma using the Mycoplasma Detection Kit (InvivoGen, Cat#: rep-mys-50) before experiments. No mycoplasma contamination was found.
Commonly misidentified lines (See ICLAC register)	No commonly misidentified cell lines were used for this study.

Animals and other research organisms

Policy information about studies involving animals; ARRIVE guidelines recommended for reporting animal research, and Sex and Gender in Research

Laboratory animals	Wide type C57BL/6 mouse line (Cat#:000664) or transgenic C57BL/6 mouse line, with EGFP cDNA (Cat#: 003291) were ordered from Jackson laboratory and housed in a specific-pathogen-free animal facility at ambient temperature (22 ± 2 °C), air humidity 40%–70% and 12-h dark/12-h light cycle.
Wild animals	No wild animal was used in this study.
Reporting on sex	Female mice were used in this study
Field-collected samples	No samples were collected in Field.
Ethics oversight	All animal experiment protocols were reviewed and approved by the institutional animal care and use committee of the University of Pennsylvania.

Note that full information on the approval of the study protocol must also be provided in the manuscript.

Clinical data

Policy information about clinical studies
All manuscripts should comply with the ICMJE guidelines for publication of clinical research and a completed CONSORT checklist must be included with all submissions.

Clinical trial registration	N/A
Study protocol	N/A
Data collection	N/A
Outcomes	N/A

Plants

Seed stocks	N/A
Novel plant genotypes	N/A
Authentication	N/A

Flow Cytometry

Plots

Confirm that:

☒ The axis labels state the marker and fluorochrome used (e.g. CD4-FITC).

☒ The axis scales are clearly visible. Include numbers along axes only for bottom left plot of group (a 'group' is an analysis of identical markers).

☒ All plots are contour plots with outliers or pseudocolor plots.

☒ A numerical value for number of cells or percentage (with statistics) is provided.

Methodology

Sample preparation	Tumor tissues were dissociated using 1 mg/ml type I collagenase with of 50 U/ml RNase and DNase I at 37°C for 40 min. Digestion mixture was passed through 70 µm cell strainers (FALCON) to obtain single-cell suspension. Red blood cells (RBC) were removed using RBC lysis buffer (BD Biosciences). Dead cells in the single-cell suspension were excluded using Live/Dead Fixable Aqua Dead Cell Stain Kit (Life Technologies).
Instrument	BD LSR II
Software	FlowJo software package (Flowjo V10)
Cell population abundance	Sorting was not used in this study
Gating strategy	In general, cells were first gated on FSC/SSC. Singlet cells were gated using FSC-H and FSC-A. Dead cells were then excluded and further surface or intracellular antigen gating was performed on the live cell population (Extended Data Fig. 5 and Extended Data Fig. 7).

☒ Tick this box to confirm that a figure exemplifying the gating strategy is provided in the Supplementary Information.
